# Visual avoidance in phobia: particularities in neural activity, autonomic responding, and cognitive risk evaluations

**DOI:** 10.3389/fnhum.2013.00194

**Published:** 2013-05-31

**Authors:** Tatjana Aue, Marie-Eve Hoeppli, Camille Piguet, Virginie Sterpenich, Patrik Vuilleumier

**Affiliations:** ^1^Swiss Center for Affective Sciences, University of GenevaGeneva, Switzerland; ^2^Department of Neuroscience, University Medical Center, University of GenevaGeneva, Switzerland; ^3^Alan Edwards Centre for Research on Pain, McGill UniversityMontreal, QC, Canada

**Keywords:** phobia, fear, cognitive risk, visual attention, vigilance-avoidance, fMRI, autonomic nervous system activity, eye tracking

## Abstract

We investigated the neural mechanisms and the autonomic and cognitive responses associated with visual avoidance behavior in spider phobia. Spider phobic and control participants imagined visiting different forest locations with the possibility of encountering spiders, snakes, or birds (neutral reference category). In each experimental trial, participants saw a picture of a forest location followed by a picture of a spider, snake, or bird, and then rated their personal risk of encountering these animals in this context, as well as their fear. The greater the visual avoidance of spiders that a phobic participant demonstrated (as measured by eye tracking), the higher were her autonomic arousal and neural activity in the amygdala, orbitofrontal cortex (OFC), anterior cingulate cortex (ACC), and precuneus at picture onset. Visual avoidance of spiders in phobics also went hand in hand with subsequently reduced cognitive risk of encounters. Control participants, in contrast, displayed a positive relationship between gaze duration toward spiders, on the one hand, and autonomic responding, as well as OFC, ACC, and precuneus activity, on the other hand. In addition, they showed reduced encounter risk estimates when they looked longer at the animal pictures. Our data are consistent with the idea that one reason for phobics to avoid phobic information may be grounded in heightened activity in the fear circuit, which signals potential threat. Because of the absence of alternative efficient regulation strategies, visual avoidance may then function to down-regulate cognitive risk evaluations for threatening information about the phobic stimuli. Control participants, in contrast, may be characterized by a different coping style, whereby paying visual attention to potentially threatening information may help them to actively down-regulate cognitive evaluations of risk.

## Introduction

Fear is an emotion that influences what is in the focus of attention and what is ignored. According to Öhman and Mineka (2001), evolution has formed highly conserved fear circuits that ensure rapid focusing of attention on potential threat sources in order to prioritize the processing of fear- or survival-relevant situations. Research has distinguished between early, automatic, and later, more controlled mechanisms of attention deployment. The most prominent view is that phobic and anxious individuals are characterized by a so-called vigilance-avoidance pattern, implying an early enhanced automatic direction of attention toward a threat source, but subsequent diversion of attention away from the threat, when more controlled processes come into play (e.g., Mogg et al., 1997; Amir et al., 1998; Rinck and Becker, 2006).

In an exemplary study, Hermans et al. (1999) simultaneously presented images of spiders and flowers to spider fearful and non-spider fearful individuals. During the first 500 ms of stimulus presentation, spider fearful and non-spider fearful individuals did not differ in their fixation times on spiders; both looked longer at spiders than they did at flowers. However, afterward, spider fearful participants avoided looking at the spiders. Thus, this study speaks to differences in later, more controlled attention deployment between the two groups of participants, but, contrary to the conceptions of Öhman and collaborators (e.g., Öhman et al., 2001), not to differences in initial vigilance. Whether speeded automatic threat detection occurs or not may depend on task characteristics (Rinck et al., 2005).

The hypothesis of avoidance during controlled processing of fear-related stimuli in highly fearful or phobic individuals is corroborated by other studies that used free viewing time (e.g., Hamm et al., 1991; Tolin et al., 1999). However, the reasons for and consequences of such viewing behavior are still unclear. Among other things, the exact conditions under which visual avoidance sets in remain to be identified. In some situations, a phobic individual visually ignores phobic stimuli, but in other situations does not. Likewise, not every phobic individual displays a similar degree of visual avoidance in a given situation.

Better knowledge of brain responses and peripheral physiology might help to uncover important mechanisms at the basis of phobic visual avoidance and thus help to refine hypotheses about the origin and function of such behavior [for the promise and limitations of functional magnetic resonance imaging (fMRI) in the study of psychological phenomena, see (Aue et al., 2009)]. Visual avoidance is often considered as a sign of a fear regulation deficit (i.e., individuals are unable to actively cope with the perceived threat because they feel their own resources do not match the situational demands; Helbig-Lang and Petermann, 2010). Such viewing behavior may be part of a de-escalation strategy that prevents the fear response from completely unfolding, thus being beneficial in the short run^1^.

We therefore hypothesized that visual avoidance tends to arise when phobic individuals feel particularly threatened and fearful. If this really were the case, we would expect visual avoidance in phobia to vary as a positive function of initial activity in the fear circuit. This would imply, among other things, increased activity within the amygdala (for the implication of the amygdala in animal phobia, see Carlsson et al., 2004; Åhs et al., 2009) and increased autonomic arousal (Sarlo et al., 2002; Mühlberger et al., 2006; Wendt et al., 2008). Such heightened amygdala and autonomic activity could be associated with the perception of increased cognitive conflict, thus enhancing the need for regulatory actions (e.g., visual avoidance).

Alternatively, it is also conceivable that avoidance behavior is negatively associated with fear level (and concomitant amygdala and autonomic activity). In fact, phobic individuals do not need to experience fear at all if they know that a threatening situation can be successfully avoided (for a discussion on emotion avoidance strategies as opposed to emotion-driven behavior, see Barlow et al., 2004). In that case, the initiation of rapid visual avoidance could prevent fear from setting in. Therefore, phobic individuals who avoid looking at potentially threatening scenes might be more successful in preventing the fear response from unfolding than those who do not.

Other brain regions that could play an important role in visual avoidance are located in the orbitofrontal cortex (OFC). Bishop (2007), for instance, suggested that altered coupling of the amygdala-prefrontal (including medial and lateral OFC) circuitry underlies fear and anxiety. Along these lines, an influential view on the regulation of negative affect sees the prefrontal cortex as a crucial site for the down-regulation of amygdala activity (Rosenkranz et al., 2003; Quirk and Beer, 2006). Contrary to the latter view, however, more recent research suggests OFC-amygdala co-activation to be responsible for successful down-regulation of negative affect (Banks et al., 2007). Although opposing, these two views point to the importance of interactions between the amygdala and the OFC in the evolvement of negative emotions such as fear.

Consistent with this observation several findings from human brain imaging studies indicate that anxiety disorders are characterized by elevated amygdala activity, on the one hand, and abnormal activity in the ventromedial prefrontal cortex (vmPFC) and/or the ventrolateral prefrontal cortex (vlPFC), on the other (for supportive evidence in animal fear, see Rauch et al., 1995; Carlsson et al., 2004; Schienle et al., 2007; Straube et al., 2007; Åhs et al., 2009). Although there is great inconsistency regarding the direction of effects in the prefrontal cortex, deviating prefrontal changes have most often been assumed to reflect fear regulation difficulties (e.g., Hermann et al., 2009). Because visual avoidance can be seen as a specific form of regulation, it can be hypothesized that the OFC (possibly in conjunction with the amygdala) is implicated in visual avoidance as well.

In the current study, we aimed to uncover both central and autonomic mechanisms at the basis of visual avoidance in spider phobia. We also wanted to determine whether eye gaze behavior (i.e., duration of fixations on spider stimuli as recorded by eye tracking) is directly related to cognitive evaluations of risk and subjective feelings of fear. We thereby hoped to shed light on the function of visual avoidance. It is, for instance, conceivable that visual avoidance of a threat source corresponds with cognitive avoidance (according to the principle “out of sight, out of mind”) and therefore leads to a reduction in risk estimation for threat encounter, as well as diminished experience of fear. However, direct evidence demonstrating such links is still missing.

While undergoing fMRI, spider phobic and non-spider phobic participants viewed pictures of spiders, snakes, and birds; estimated the risk that they would encounter these animals at different forest locations (cognitive evaluation); and rated their fear intensity (subjective feeling). During task performance, the participants' eye fixations as well as their central and autonomic nervous system responses (heart rate and skin conductance) were recorded.

In sum, we hypothesized that (1) spider phobic participants would be characterized by visual avoidance of spiders; (2) such avoidance would vary as a positive function of activity in the fear circuit (with characteristic central and autonomic activations); and (3) these increases would be accompanied by altered activity in the OFC. We further predicted that greater visual avoidance in spider phobia would be associated with lowering of the generally increased (4) cognitive evaluations of personal risk, and (5) subjective fear levels (Aue and Hoeppli, 2012). Moreover, we expected these predicted associations (points 2–5) to be qualitatively different from those observed for spiders in the control group (i.e., to be specific for spider phobia). We further investigated this idea of phobia-specific associations by including responses to snakes (that neither spider phobics nor controls feared) in statistical testing; no differences in associations between the two groups were expected for these animals.

## Materials and methods

### Participants

Participants were recruited via advertisements placed in university buildings and on regional advertisement websites. Individuals interested in the study were interviewed by telephone and assessed with the *Diagnostic and Statistical Manual of Mental Disorders* (4th ed., text revision; American Psychiatric Association, 2000) and the *International Classification of Diseases* (10th revision; World Health Organization, 1992) criteria for the presence or absence of spider phobia and comparably low fear of snakes (adapted from Mühlberger et al., 2006). Thirty-six right-handed individuals (all female, 18 spider phobics), aged between 19 and 44 years (*M* = 25.8, *SD* = 5.79), without history of neurological illness and use of neuroleptics, anxiolytics, or antidepressants, took part in the study. One participant in the phobic group was excluded because of problems with eye gaze acquisition, resulting in an insufficient number of valid eye-tracking data samples (<30%). An additional participant in the control group was exempted because she had not performed the task correctly.

During the telephone interview, participants rated their fear of spiders and snakes on a scale from 0 (*no fear at all*) to 100 (*maximal or extreme fear*). Spider phobic participants rated their fear of spiders higher than did control participants, *t*_(32)_ = 14.76, *p* < 0.000001 (*M*s = 83.5 and 16.4). The two groups did not differ with respect to their (low) ratings for fear of snakes, *t*_(32)_ = −0.27, *ns* (*M*s = 11.5 and 12.4). Fear of spiders and snakes was also assessed after the experiment by the use of the fear of spiders questionnaire (Szymanski and O'Donohue, 1995), *t*_(32)_ = 8.95, *p* < 0.000001 (*M*s = 86.4 and 23.5), and the Snake Questionnaire (Klorman et al., 1974), *t*_(32)_ = 0.74, *ns* (*M*s = 4.1 and 3.2). Participants in the two groups did not differ in age, *t*_(32)_ = −0.42, *ns* (*M*s = 25.1 and 25.9).

### Stimuli

Stimuli consisted of 30 pictures displaying spiders and 30 pictures displaying snakes (taken from the Geneva Affective PicturE Database; Dan-Glauser and Scherer, 2011). Spider and snake pictures were matched for valence, *t*_(58)_ = 0.08, *ns* (*M*s = 3.1 and 3.1; *SD*s = 0.94 and 0.95, for spiders and snakes, respectively; scale range: 1 [*very unpleasant*]—9 [*very pleasant*]); and arousal ratings, *t*_(58)_ = 0.03, *ns* (*M*s = 6.1 and 6.1; *SD*s = 0.88 and 0.75, for spiders and snakes, respectively; scale range: 1 [*not arousing at all*]—9 [*very arousing*]), as assessed in an earlier study (Dan-Glauser and Scherer, 2011) with an unselected group of undergraduate students. Thirty additional pictures displaying birds were collected from the Internet. Pictures of 10 neutral animals (e.g., goats and frogs) were included for use in 10 practice trials.

### Setting and apparatus

MRI data were acquired from a 3T scanner (Trio TIM, Siemens, Germany) with the product 12-channel head coil. Autonomic nervous system activity was acquired continuously with the Biopac MP150 System (Goleta, CA, USA). There were different settings for the electrocardiogram and skin conductance channels (see section Autonomic Nervous System Data, for details)^2^. Autonomic signals were transferred from the experimental room to the MP150 Acquisition Unit (16 bit A/D conversion) in the control room and stored on computer hard disk. A digital channel received inputs from the presentation computer and recorded on- and offset of the presented stimuli.

Visual stimuli were presented on a back projection screen inside the scanner bore using an LCD projector (CP-SX1350, Hitachi, Tokyo, Japan). Participants' eye movements were monitored continuously at a sampling rate of 60 Hz with the EyeTrac6 Eye Tracking System (Applied Sciences Laboratories, Bedford, MA, USA). The eye camera is characterized by easily accessible focus and iris adjustments. The illuminator source is an FCR lamp (12 VDC power supply; non-coherent illumination). Eye irradiance was less than 0.5 mW/cm^3^.

Behavioral responses were recorded with a response button box (HH-1 × 4-CR, Current Designs, Inc., Philadelphia, PA, USA). Experimental control was performed by E-Prime 2 Professional (Psychology Software Tools, Sharpsburg, PA, USA).

### Procedure

Upon the participants' arrival at the laboratory, the nature of the experiment was explained and written informed consent was obtained in accordance with the Helsinki Declaration of Human Rights (World Medical Association, 1999) and regulations of the local ethics committee. Before the start of the experiment, participants performed 10 practice trials and a standardized calibration procedure for eye movements was undertaken. During this procedure, participants looked at 9 dots appearing at different locations on the computer screen.

**Figure 1:**
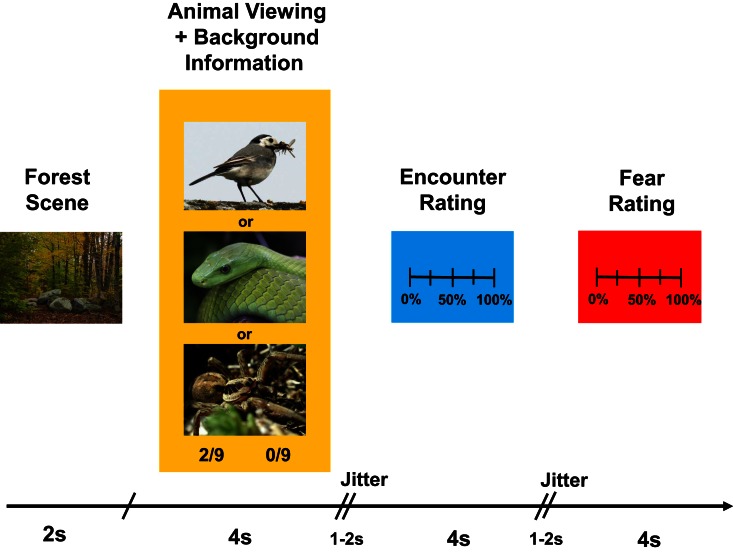
**Timeline of an experimental trial.** See text for more details.

In the experimental task, they imagined visiting different forest locations at which two forest officials had encountered specific animals before. Specifically, in each trial, participants saw a fixation cross (500 ms), followed by a picture of a forest location (1 s), followed by a picture of an animal (spider, snake, or bird; 4 s; see Figure 1). At the time they saw the animal (covering ~40% of the screen), participants simultaneously received background information about (1) the number of times the first forest official had encountered a specific animal out of the number of times he had visited the location (e.g., 2/9); and (2) the number of times the second forest official had encountered this animal out of the number of times he had visited the same location (e.g., 0/9). This background information was displayed below the pictures. Importantly, the objective probabilities (i.e., the average of the two likelihoods given as background information) were equal across the three animal categories.

From the background information, participants rated the risk that they would encounter the animal if they were themselves at that same forest location, and the fear they experienced when imagining this scenario [17-point scale ranging from 0% (*no risk of encounter at all; no fear at all*) to 100% (*absolute certainty of encounter; extreme, paralyzing fear*)]. Responses were given by pressing two buttons of a button box, which moved a slider across the scales. The time for a response was limited to 4 s for each rating.

The 90 experimental trials were presented in random order in two runs of 23 trials and two runs of 22 trials, separated by short pauses. In addition, the whole sequence was presented in a jittered manner (two jitters/random time intervals ranging between 1 and 2 s, inserted between animal/background presentation and encounter risk rating, and between encounter risk rating and fear rating), making an intertrial interval of ~15–16 s (Figure 1).

### Variables

#### Gaze duration

Participants' eye movements (i.e., gaze durations on different locations of the back projection screen) were acquired in the animal viewing/background presentation phase (see Figure 1).

#### Central nervous system data (fMRI)

Structural images were acquired with a *T*_1_-weighted 3D sequence (MPRAGE, TR/TI/TE = 1900/900/2.27 ms, flip angle = 9°, PAT factor = 2, voxel dimensions: 1 mm isotropic, 256 × 256 × 192 voxels). Functional images were acquired with a *T*_2^*^_-weighted EPI sequence (TR/TE = 2000/30 ms, flip angle = 80°, PAT factor = 2, 64 × 64 pixels, 3.2 × 3.2 mm, 36 slices, 3.2-mm slice thickness, 20% slice gap). An automatic shimming procedure was performed to minimize inhomogeneities of the static magnetic field. At the beginning of each session, image acquisition started after the recording of three dummy volumes to avoid *T*_1_ saturation effects.

MRI data were preprocessed and analyzed using SPM8 (Wellcome Department of Imaging Neuroscience, London, UK; http://www.fil.ion.ucl.ac.uk/spm). Functional images were reoriented to the AC-PC line, spatially realigned to the first volume by rigid body transformation, corrected for time differences in slice acquisition using the middle slice in time as reference, spatially normalized to the standard Montreal Neurological Institute EPI template, resampled to an isotropic voxel size of 3 mm, and spatially smoothed with an isotropic 8-mm full width at half-maximum (FWHM) Gaussian kernel (Friston et al., 1995).

#### Autonomic nervous system data

Autonomic signals were recorded continuously with a sampling rate of 10000 Hz and pre-processed with AcqKnowledge 4.1 (Biopac, Santa Barbara, CA, USA) and PPP 7.12 (Extra Quality Measurement Systems, Frankfurt am Main, Germany).

***Heart rate***. Heart rate (in beats per minute) was recorded with ConMed Cleartrace (ConMed Corporation, Utica, NY, USA) pre-gelled disposable Ag/AgCl electrodes, fixed according to Einthoven II. Amplification: 500, online high-pass filter: 0.5 Hz, offline comb band stop filter: 17.5 Hz (with all harmonics out to Nyquist; to eliminate scanner noise).

***Skin conductance***. Electrodermal activity was measured with a constant voltage of 0.5 V, using MR-compatible ConMed Cleartrace pre-gelled disposable Ag/AgCl electrodes. The transducers were placed at the volar surfaces of the medial phalanges of the index and middle fingers of the left hand. Amplification: 5 μS/V, online filters: DC and 10 Hz, offline low-pass filter: 1 Hz.

#### Rating data

Participants' encounter risk and fear ratings were registered for each experimental trial.

### Data analysis

#### Gaze duration

Missing signals in the eye-tracking data were eliminated (10–15% of all samples, due to eye blinks and signal loss). The percentage of samples spent in the region of the screen where the picture was displayed relative to the overall number of samples acquired was calculated for each participant and trial. Participants' gaze duration was then subjected to an analysis of variance with the factors Animal (spider, snake, bird) and Group (spider phobic, control). In order to investigate shifts in visual attention over time, we added the factor Time for gaze duration analyses (8 0.5 s intervals, corresponding to the 4 s of animal/background presentation time).

#### Link between gaze duration and neural responses (fMRI)

Statistical analysis was performed using the general linear model for event-related designs in SPM8. Hemodynamic response functions with 10 regressors were estimated for the whole time series: one regressor for the forest picture onset, three different regressors for the animal/background presentation onset (spider, snake, bird), three regressors for the encounter risk rating phase (same event categories as for animal/background presentation phase), and another three regressors for the fear rating phase (same event categories). Six motion-correction parameters were also added to the model. A high-pass filter of 128 s was applied to account for low-frequency noise of the scanner and first-order autoregressive corrections for autocorrelation between scans. Effects at each brain voxel were estimated using a least squares algorithm. Our analysis focused on activation patterns correlating with gaze duration to spiders (and snakes) in the two groups of participants (see below)^3^.

***Whole-brain analysis***. We performed a parametric analysis to identify brain mechanisms associated with visual avoidance in spider phobia. Because we considered birds as a neutral reference category, mean gaze duration for birds was subtracted from mean gaze duration for spiders (snakes) in each participant. The so-calculated behavioral gaze duration contrast variable was then used as a between-subjects covariate for the prediction of the BOLD contrast “spider–bird” (“snake–bird”) in a second-level group analysis. Specifically, we identified group differences in covariation effects with a second-level *t*-test. In order to avoid alpha inflation, we report only significant clusters containing at least 22 contiguous voxels at *p* < 0.001. This minimum cluster size was calculated by a Monte Carlo simulation with 10,000 iterations, assuming some interdependence between voxels (8-mm FWHM), resulting in a corrected whole-brain *p*-value of 0.01.

For the so-identified clusters, mean individual activations for spiders (snakes) and birds were extracted and the BOLD contrast “spider-bird” (“snake-bird”) was calculated. Next, correlations of the BOLD contrast and the behavioral gaze duration contrast variable were calculated separately for each group and each cluster. These group Pearson product-moment correlations were then transformed into Fisher's *Z*-values. Finally, we performed a *t*-test for independent groups to determine the significance of the observed group differences. All parametric maps were rendered on sections of the average *T*_1_-weighted template brain of the entire group (all participants).

***Regions of interest (ROIs)***. From earlier literature (e.g., Bishop, 2007), we hypothesized altered activity in the amygdala-prefrontal (more specifically OFC) circuitry to be implicated in phobic visual avoidance. Parameter estimates for amygdala and OFC—describing the mean activity change provoked by the animal picture presentation—were extracted for each participant by applying masks according to the automated anatomical labeling approach of activations (Tzourio-Mazoyer et al., 2002). The BOLD contrast “spider–bird” (“snake–bird”) was then calculated and correlated with the behavioral gaze duration contrast variable “spider–bird” (“snake–bird”), both within the phobic group and within the control group. Next, these group Pearson correlations were transformed into Fisher's *Z*-values. Finally, we performed a *t*-test for independent groups to test whether the relationship between neural activity in the ROIs and gaze duration varied as a function of experimental group (phobic vs. control).

#### Link between gaze duration and autonomic responses

We hypothesized phobic visual avoidance to vary as a positive function of autonomic arousal. Outliers [>3 *SD* from the mean value of a given participant in a given autonomic measure (heart rate; skin conductance)] and artifacts were eliminated (~1%). To obtain autonomic changes resulting from the presentation of the different stimuli, baseline scores (2-s interval before animal/background presentation phase) were subtracted from task scores in the animal/background presentation phase [heart rate: animal picture onset to picture offset; skin conductance: animal picture onset +1 s to picture offset +1 s (because skin conductance changes only slowly)].

Because of data recording problems, one phobic participant was excluded from all autonomic analyses. Because of changes in module calibration, two other participants (one phobic and one control) were excluded from skin conductance analyses. Finally, given the difficulty in obtaining a high-quality electrocardiogram in an MRI scanner, four phobic and three control participants were excluded from heart rate analyses.

For both measures (heart rate and skin conductance), the contrast “spider–bird” (“snake–bird”) was calculated and correlated with the behavioral gaze duration contrast variable “spider–bird” (“snake–bird”), both within the phobic group and within the control group. Subsequent steps were similar to those described for fMRI analyses.

#### Link between gaze duration, encounter risk ratings, and fear ratings

We wanted to know whether phobic visual avoidance would impact (i.e., decrease) behavioral ratings of encounter risk for and fear of spiders and whether this relationship would be specific for spiders in spider phobics (i.e., not present in controls and not observable for snakes in the phobic group). For the phobic and the control group, we therefore separately calculated paired Pearson product-moment correlation coefficients between the behavioral gaze duration contrast variable “spider–bird” (“snake–bird”), on the one hand, and “spider–bird” (“snake–bird”) difference scores for both encounter risk and fear ratings, on the other. Subsequent steps were similar to those described for fMRI analyses.

## Results

### Gaze duration

**Figure 2:**
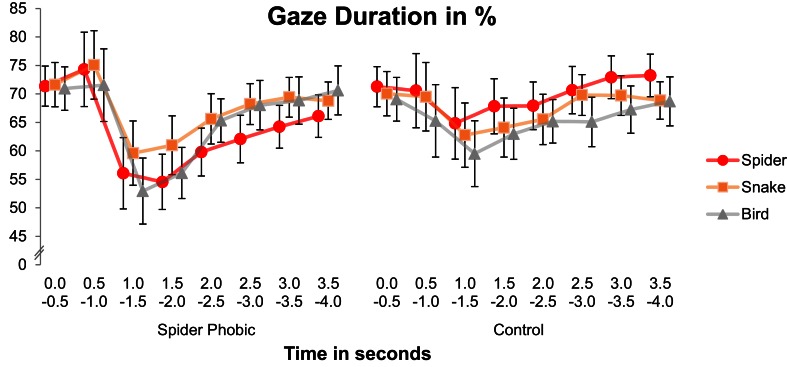
**Gaze duration on the pictures presented as a function of animal, time, and group.** Error bars depict standard errors.

Spider phobics were characterized by a visual avoidance pattern for spiders, whereas the non-fearful controls displayed a vigilance pattern (Figure 2), interaction Animal × Group, *F*_(2, 64)_ = 5.71, *p* < 0.01 [main effect of Group, *F*_(1, 32)_ = 0.15, *ns*; main effect of Animal, *F*_(2, 64)_ = 1.75, *ns*]. When the two groups were analyzed separately, the main effect of Animal failed to reach significance in the spider phobic group, *F*_(2, 32)_ = 2.18, *ns*, but the interaction Time × Animal was significant, *F*_(14, 224)_ = 1.92, *p* < 0.05. The avoidance pattern in phobics arose between 2 and 3 s following stimulus onset, as indicated by analyses of variance with the factor Animal conducted separately for each time interval, *F*s_(2, 32)_ = 3.44 and 3.05, *p*s < 0.05 and 0.07, for 2–2.5 s and 2.5–3 s, respectively. *Post-hoc* Tukey tests revealed that, in both cases, gaze durations for spiders were (marginally) shorter than gaze durations for both snakes and birds (all *p*s for corresponding pairwise comparisons <0.11). At the same time, no difference in gaze duration for snakes vs. birds was observed in this group (*p*s > 0.99). For controls, the main effect of Animal turned out to be significant, *F*_(2, 32)_ = 7.67, *p* < 0.005, but not the interaction Time × Animal, *F*_(14, 224)_ = 0.59, *ns*. They consistently looked longer at spiders than at birds (*p* < 0.005; remaining pairwise comparisons: *p*s > 0.11)^4^.

### Link between gaze duration and neural responses

#### Whole-brain analysis

**Figure 3:**
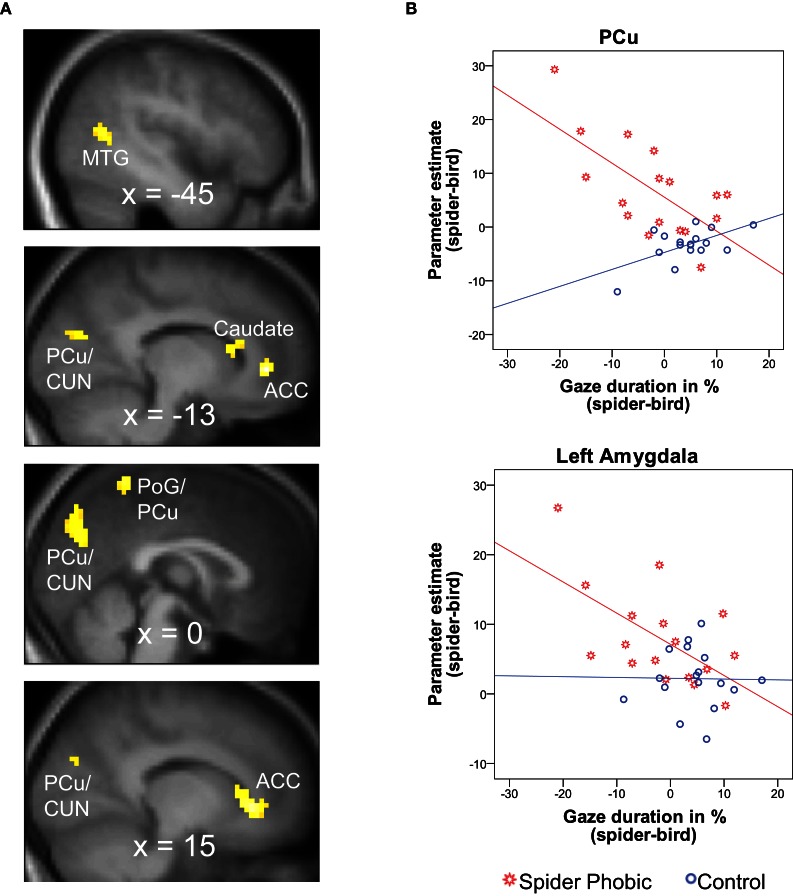
**Brain areas demonstrating a differential association between gaze duration_spider–bird_ and BOLD activation_spider–bird_ in spider phobics vs. controls. (A)** Significant clusters in whole-brain analysis; negative association in phobics; positive association in controls. **(B)** Illustration of the effects for the precuneus (largest cluster in whole-brain analysis) and the left amygdala (ROI analysis). ACC, anterior cingulate gyrus; CUN, cuneus; MTG, middle temporal gyrus; PCu, precuneus; PoG, postcentral gyrus; ROI, region of interest.

The whole-brain parametric analyses based on gaze duration yielded six clusters, whose activation pattern for spiders (vs. birds) was differently related to gaze duration (difference in gaze duration for spiders vs. birds) in the two experimental groups. Two clusters were located in the anterior cingulate cortex (ACC), and others were in the precuneus/cuneus, the medial postcentral gyrus/precuneus, the caudate, and the middle temporal gyrus. In all cases, phobics demonstrated a negative association between gaze duration and activation, whereas it was the reverse for controls (see Figure 3; Table 1). A similar whole-brain analysis conducted for snakes (vs. birds) did not yield any group difference, thus showing that the above-described associations in spider phobics were specific to phobogenic stimulus material.

**Table 1:** **Correlations between gaze duration _spider–bird_ and BOLD contrast_spider–bird_; whole brain analysis**.

**Region**	**CS**	**MNI coordinates**			***r*_Phobic_**	***r*_Control_**	***Z***
		***x***	***y***	***z***			
ACC	31	−12	41	−2	**−0.81**	*0.42*	**−4.17**
ACC	76	15	32	−5	**−0.72**	**0.65**	**4.45**
Precuneus/cuneus	169	−3	−76	28	**−0.67**	**0.59**	**3.94**
Caudate	81	−13	18	12	**−0.70**	**0.67**	**4.44**
Postcentral gyrus/precuneus	25	0	−49	64	**−0.64**	**0.58**	**3.76**
Middle temporal gyrus	32	−45	−61	10	**−0.56**	**0.74**	**4.19**

N = 17 in each group. Bold: p < 0.05 (two-tailed); italics: p < 0.10 (two-tailed). ACC, anterior cingulate cortex; CS, cluster size in number of voxels; MNI, Montreal Neurological Institute; Z, Fisher's Z transformation, testing the difference between the two group correlations.

#### ROI analysis

In accordance with our hypotheses, phobics demonstrated a negative association between gaze duration for spiders (difference in gaze duration for spider vs. bird) and BOLD activation to spiders (vs. birds) in bilateral amygdala (Figure 3). The same association was observed in the OFC. Controls, on the other hand, did not show any significant association between gaze duration for spiders and BOLD activity in any of the investigated ROIs (Table 2). Again, these associations originated from phobia-specific responses to spiders in spider phobics; we did not find any group difference when contrasting associations for snakes vs. birds.

**Table 2:** **Correlations between gaze duration _spider–bird/snake–bird_ and BOLD contrast _spider–bird/snake–bird_; ROI analysis**.

		**Spider–Bird**			**Snake–Bird**		
		***r***_**Phobic**_	***r***_**Control**_	***Z***	***r***_**Phobic**_	***r***_**Control**_	***Z***
Amygdala	L	**−0.60**	−0.02	−*1.78*	−0.11	0.30	−1.11
	R	**−0.49**	0.16	−1.07	−0.18	0.25	−1.16
**OFC**							
Gyrus rectus	L	**−0.60**	−0.04	−*1.73*	−0.07	−0.15	0.21
	R	**−0.72**	−0.34	−1.46	−0.11	−0.16	0.13
F1O	L	**−0.57**	0.30	−**2.53**	−0.03	0.07	−0.26
	R	**−0.59**	−0.09	−1.55	−0.04	0.43	−1.32
F1MO	L	**−0.52**	0.14	−*1.90*	−0.11	0.12	−0.61
	R	**−0.71**	0.08	−**2.56**	−0.12	0.13	−0.66
F2O	L	−*0.43*	−0.08	−1.00	0.09	0.27	−0.49
	R	−0.23	−0.17	−0.17	0.03	0.24	−0.57
F3O	L	**−0.57**	0.00	−*1.71*	0.07	0.35	−0.78
	R	*−0.45*	0.18	−*1.76*	0.01	**0.53**	−1.53

N = 17 in each group. Bold: p < 0.05 (two-tailed); italics: p < 0.10 (two-tailed); ROI, region of interest; OFC, orbitofrontal cortex; F1O, superior frontal gyrus, orbital part; F1MO, superior frontal gyrus, medial orbital; F2O, middle frontal gyrus, orbital part; F3O, inferior frontal gyrus, orbital part, as defined by the automated anatomical labeling of activations in SPM by Tzourio-Mazoyer et al. (2002); L, left; R, right. Z, Fisher's Z transformation, testing the difference between the two group correlations.

### Link between gaze duration and autonomic responses

Shorter gaze duration for spiders in spider phobics was associated with increased autonomic arousal, and the opposite association was observed in controls (Table 3). Both the associations for heart rate and skin conductance show phobia specificity^5^.

**Table 3:** **Correlations between gaze duration _spider–bird/snake-bird_ and autonomic responses _spider–bird/snake-bird_**.

	**Spider–Bird**			**Snake–Bird**		
	***r***_**Phobic**_	***r***_**Control**_	***Z***	***r***_**Phobic**_	***r***_**Control**_	***Z***
Heart rate	−0.44	0.48	**−2.21**	0.18	0.35	−0.41
Skin conductance	−0.25	0.78	**−3.04**	0.49	0.43	0.18

Heart rate: *N*_Phobic_ = *12*, *N*_Control_ = *14*; Skin conductance: *N*_Phobic_ = *13*, *N*_Control_ = *15*. Bold: p < 0.05 (two-tailed). Z, Fisher's Z transformation, testing the difference between the two group correlations.

### Link between gaze duration, encounter risk rating, and fear rating

Differential gaze duration for spiders vs. birds was not significantly related to differential risk ratings for spiders vs. birds in either phobics or controls (same for snakes vs. birds). However, in a more general manner, phobics displayed a positive association between gaze duration and encounter risk estimates for all animals (Table 4). Thus, the more intensely the images were inspected, the higher the later encounter risk ratings. In controls, the reverse was true. Fear ratings were altogether unrelated to gaze duration.

**Table 4:** **Correlations between gaze duration and participants' encounter risk and fear ratings**.

	**Gaze duration**								
	**Spider vs. Bird**			**Spider**			**Bird**		
	***r***_**Phobic**_	***r***_**Control**_	***Z***	***r***_**Phobic**_	***r***_**Control**_	***Z***	***r***_**Phobic**_	***r***_**Control**_	***Z***
**ENCOUNTER RISK RATING**									
Spider vs. Bird	0.29	−0.08	1.00						
Spider				*0.46*	**0.74**	**3.83**			
Bird							*0.43*	**0.61**	**3.09**
**FEAR RATING**									
Spider vs. Bird	−0.02	0.40	1.17						
Spider				0.29	0.01	0.76			
Bird							0.03	0.03	0.00
	**Snake vs. Bird**			**Snake**					
	***r***_**Phobic**_	***r***_**Control**_	***Z***	***r***_**Phobic**_	***r***_**Control**_	***Z***			
**ENCOUNTER RISK RATING**									
Snake vs. Bird	0.24	−0.09	0.89						
Snake				*0.45*	**0.76**	**3.92**			
**FEAR RATING**									
Snake vs. Bird	0.15	0.11	0.11						
Snake				0.16	0.12	−0.75			

N = 17 in each group. Bold: p < 0.05 (two-tailed); italics: p < 0.10 (two-tailed). Z, Fisher's Z transformation, testing the difference between the two group correlations.

## Discussion

### Gaze duration

Our results are only partly consistent with the hypothesis of a vigilance-avoidance pattern of visual attention for feared content in specific phobia (Mogg et al., 1997; Amir et al., 1998). We did not find increased vigilance for (i.e., increased gaze durations to) phobic stimuli in the early phase of picture presentation in the phobic group. However, Figure 2 shows that all of our participants slightly enhanced their gaze duration for both spiders and snakes as compared with birds at around 0.5 s following stimulus onset. This enhancement may have been too rapid or short-lasting to reach statistical significance. Thus, even if this effect is not significant, the curve fits with the hypothesis of early vigilance for fear-relevant stimuli (yet, there definitely did not seem to be a surplus of initial vigilance to phobic content).

More importantly, we observed an avoidance pattern of gaze duration for spiders in spider phobics at a later, more controlled processing stage, starting at about 2 s after stimulus onset (cf. Hermans et al., 1999). Control participants, on the other hand, looked longer at spiders than at birds, consistent with the idea that biological threat generally attracts attention in normal controls (e.g., Öhman and Mineka, 2001; Vuilleumier, 2005). Hence, our results do not favor such a “normal” negativity bias to exist in phobic individuals^6^.

### Link between gaze duration and neural responses

Spider phobic participants displayed substantially different associations between neural responses and gaze duration for spiders than did control participants in our study. Moreover, the fact that we observed no differential association of brain responses and viewing behavior for snakes in spider phobics vs. controls underscores that the associations in spider phobics were specific to phobic content.

We found that phobic participants who were characterized by particularly strong amygdala activation to spiders upon picture onset were also those who demonstrated particularly strong visual avoidance of spiders. The amygdala projects to regions that control arousal and orienting to sensory stimuli (e.g., nucleus basalis, hypothalamus, thalamus) and therefore can exert considerable influence on defensive perceptual and attentional processes (e.g., Armony and Dolan, 2002; Hamm and Weike, 2005), even before sensory information reaches conscious awareness (LeDoux and Phelps, 2000). These data are therefore consistent with our hypothesis that heightened activity in the fear circuit is at the origin of visual avoidance in spider phobia. Controls, in contrast, did not show any association of spider-related activity in the amygdala and gaze duration. However, this is not necessarily surprising, because our control participants were selected because of their low fear of spiders.

Moreover, in parallel to amygdala activity, somatosensory cortex (postcentral gyrus) activity in response to spiders was also negatively related to gaze duration in phobics^7^. The somatosensory cortex has previously been reported to be implicated in the experience of fear and phobia (Rauch et al., 1995; Sehlmeyer et al., 2009).

In addition, we observed higher caudate activity to be associated with increased visual avoidance behavior for spiders in spider phobics (but increased vigilance in controls). A recent neuroimaging study related threat anticipation in humans to elevated caudate activity (Choi et al., 2012). Mogenson et al. (1980) proposed that the caudate is an important structure in the translation of motivational states into behavioral action. Support for such an interpretation comes from studies demonstrating that lesions to the caudate prevent avoidance learning or the initiation of avoidance behavior in animals (e.g., Winocur and Mills, 1969) and that activity in this area is related to the personality trait, behavioral inhibition (Helfinstein et al., 2012). What is more, the head of the caudate nucleus is intimately linked to neural pathways connecting prefrontal cortical areas that control eye movements (e.g., frontal eye fields) with subcortical oculomotor centers, such as the superior colliculus (e.g., Petit et al., 1996; Lynch and Tian, 2006; Harsay et al., 2011). Thus, the initiation of gaze avoidance in spider phobia may emanate from increased activity in the caudate and subsequent projections to oculomotor pathways.

Taken together, these observations converge to suggest that visual avoidance in phobia (and vigilance in less fearful individuals) sets in *after* the fear circuit has already been activated, and not *before*. Therefore, it is likely that visual avoidance, in our case, did not prevent the emergence of the fear response altogether. Rather, our findings may indicate that greater fear leads to stronger avoidance.

Other differences between the two groups of participants were found in a number of brain regions that have been related to (attempts at) emotion regulation in earlier research, namely, the OFC, ACC, and precuneus (e.g., Botvinick et al., 2001; Cavanna and Trimble, 2006; for activations with respect to animal phobia, see Rauch et al., 1995; Carlsson et al., 2004; Schienle et al., 2007; Straube et al., 2007; Hermann et al., 2009; for an implication of these areas in anxiety disorders, in general, see Charney, 2003). Whereas phobics displayed a negative association between activity in the ACC and precuneus, on the one hand, and gaze duration to pictures of spiders, on the other, controls displayed a positive association. In addition, the OFC was also negatively related to gaze duration for spiders in phobics but unrelated to gazing behavior in controls.

In line with their supposed importance for emotion regulation processes, the OFC, ACC, and precuneus have also been found to be implicated to various degrees in stimulus-driven orienting, attention, salience, and self-relevance (Botvinick et al., 2001; Cavanna and Trimble, 2006; Sturm et al., 2006), as well as in interoception and control of autonomic arousal (Critchley et al., 2004). In sum, our brain data therefore add support to the idea that the more fear-evoking and personally salient the phobia-related material is experienced, the more the phobic participants will feel themselves unable to actively cope with the situation and thus unable to continue looking at the spiders. This may in turn trigger regulatory actions (i.e., visual avoidance) with immediate adaptive benefits.

Interestingly, though, Hermann et al. (2009) and Schienle et al. (2007) related reduced activity in the vmPFC, including the medial OFC, to *reduced* automatic regulation capacities in spider phobia^8^. In addition, Hermann et al. (2009) found rostral ACC activity to be reduced in effortful down-regulation of fear of spiders. Because these authors used a within-subjects approach, whereas we used a between-subjects approach, it is difficult to directly compare our results with theirs. However, there are also some other important differences between these two studies and our own. First, we performed correlational statistics and related brain activity to gaze duration; and second, visual avoidance in our study was an acceptable response (participants just paid greater attention to the background information without completely disengaging from the task). By contrast, in Hermann et al.'s (2009) study, visual avoidance would have meant disengaging completely from the task, and thus was an explicitly undesired form of regulation.

Unlike spider phobics, our controls displayed vigilance toward spiders and a positive association of gaze duration for spiders and ACC and precuneus activity. Thus, for them, heightened activity in the regulation network may be associated with increased scanning of the environment and increased alertness. Because controls were characterized by low fear of spiders, they must have felt capable of actively coping with the situation. The strongest need for regulation and the strongest attempt at regulation may therefore be expected to correspond with increased gaze duration in this group of participants. Finally, in line with our own study results, van Reekum et al. (2007) observed that brain activity during emotion regulation co-varied with gaze behavior and that the middle temporal gyrus and cuneus were activated when their (unselected, non-phobic) participants down-regulated their negative emotions in response to presented pictures.

### Link between gaze duration and autonomic responses

Autonomic nervous system data further strengthen our hypothesis that activity in the fear circuit triggers visual avoidance of phobic content. We observed that higher autonomic arousal during the presentation of spider pictures in spider phobic participants was associated with shorter gaze durations on the pictures. Such avoidance behavior might, in turn, contribute to down-regulating autonomic arousal and the associated experience of threat. In controls, in contrast, increased arousal, as a potential indicator of fear circuit activity, corresponded with increased vigilance.

Additional analyses for snakes demonstrated that the association of autonomic arousal and gaze behavior for spiders in spider phobia was phobia specific because we did not observe group differences for snakes. However, it is important to note that correlations for spiders in spider phobics were altogether quite low (and did not significantly deviate from 0). Therefore, these links need to be replicated with a larger number of participants.

### Link between gaze duration, encounter risk rating, and fear rating

We found cognitive evaluations of encounter risk to vary as a combined function of population and gaze duration. Specifically, in spider phobics, the longer the animals (spiders, snakes, and birds) were directly looked at, the higher these participants estimated the risk to encounter them. Conversely, in controls, encounter risk estimation decreased as a linear function of gaze duration (again for all animals). Thus, cognitive processing related to visual attention was fundamentally different in phobics than in controls.

Our rating data suggest that one reason for phobics to avoid phobic (and non-phobic) information may be to down-regulate cognitive risk evaluations. Continued looking at spider pictures in phobics may make the situation more real to them and provoke escalating thoughts of danger or catastrophic thinking. One way to avoid that would be to quickly look away from the stimulus. Thus, we speculate that visual avoidance triggers a process of cognitive avoidance in spider phobia that counteracts escalating thoughts. In contrast, controls may have focused more on specific animal characteristics that made them judge encounters as unlikely, despite the “objective” background information that was given to them via the forest officials. In their case, vigilance may have helped to down-regulate cognitive risk.

Unexpectedly, we did not observe any significant group difference for the link between visual attention and subjective feelings of fear. Both the fear ratings of the phobic and of the control group did not vary as a linear function of earlier visual attention on the pictures presented. Perhaps there were lower correlations of gaze duration and subsequent fear ratings because the fear ratings always happened later than the encounter risk ratings (recall that the onset of the risk rating was about 5 s before the onset of the fear rating). It is also possible that visual avoidance in the initially more fearful phobic participants may have successfully reduced experienced fear in the meantime, whereas those who attended to the threat thereby increased their fear level.

### Integrative model of visual avoidance behavior in spider phobia

We postulate that initial activity in the fear circuit (e.g., amygdala, together with the associated autonomic arousal) can provide signals of personal salience and potential threat to individuals, thus triggering sensations of conflict (ACC) with respect to the goal of personal safety. This leads to subsequent regulation attempts, in our specific case, visual avoidance (mediated by a network of areas in the caudate, OFC, and precuneus)^9^.

But why do spider phobics use visual avoidance; what is its function? Compared with controls, spider phobic participants were characterized by markedly higher cognitive evaluations of risk for encounters with spiders (Aue and Hoeppli, 2012). The present article extends these previous results by showing that this overestimation of cognitive risk was positively related to gaze duration. We hypothesize that vigilance to threatening pictures in phobic participants triggers a fight-flight response that is accompanied by escalating thoughts of negative consequences, increased autonomic arousal, and the subjective experience of fear. Because phobic individuals are not able to actively (i.e., cognitively) disengage from this escalation of threatening thoughts when they continue looking at spiders, they can only reduce their mental and physiological arousal, as well as their subjective fear, by looking away from spiders.

Importantly, our model primarily holds for confrontation with spiders in the laboratory context (e.g., when only images of feared animals are presented). Meeting spiders in a real-life situation could provoke dramatically different behavior because phobic individuals know that this type of danger cannot be suppressed if it is only visually avoided (cf. the defense cascade model; Bradley and Lang, 2007). According to their own beliefs, such ignorance could have fatal consequences (e.g., “the spider will touch or bite me”). Therefore, in real-life threatening situations, we would expect gaze behavior in phobics to take the opposite direction. As long as there is no way to distance herself physically from the threatening object, a phobic individual will probably pay particularly strong visual attention to the scene and thus may show quantitatively different (exaggerated) responses relative to control individuals (rather than qualitatively different responses, as revealed in the current study).

### Limitations

We cannot be certain about the causal direction of the correlation effects we assumed because we did not independently manipulate gaze duration. For instance, rather than having cognitive evaluations being influenced by visual attention, the impression of high encounter risk could ensure that a stimulus is attended to. However, if this really were the case, we would have expected spider phobics to look *longer* at spiders than at snakes and birds because their encounter risk estimates were generally higher for spiders than for both snakes and birds (for details, see Aue and Hoeppli, 2012). Instead, the reverse effect was observed about 2 s after stimulus onset (visual avoidance of spiders), which therefore speaks against considering phobic visual avoidance as a consequence rather than a precedent of elevated cognitive risk evaluations.

Similarly, because of the pure correlational nature of our findings, we are not safe in saying that the characteristic neural activities in our study are precursors of avoidance and vigilance behavior in spider phobics vs. controls. Alternatively, neural effects observed here might be a mixture of causes and consequences. Future studies are needed to further investigate the direction of these effects. These studies should also increase the number of valuable datasets for autonomic arousal measures.

### Conflict of interest statement

The authors declare that the research was conducted in the absence of any commercial or financial relationships that could be construed as a potential conflict of interest.

## Footnotes

^1^Despite positive short-term effects, it is also evident that such avoidance behavior is likely to prevent the experience of habituation processes and, in the long run, a realistic evaluation of the situation.

^2^Respiration rate and muscle activity over the cheek and brow regions (measured over the M. Zygomaticus major and the M. Corrugator supercilii, respectively) had also been assessed. Because of space limitations and because they are not of central importance for the current investigation, these variables will not be specified. For further details, see Aue et al. (2012).

^3^Analyses for snakes were included to test the idea that associations between gaze duration and neural activations/deactivations for spiders in spider phobics are phobia specific. In contrast to the analyses for spiders, no differences in associations between spider phobics and controls were expected for snakes.

^4^In both spider phobics and controls, the extent of visual avoidance/vigilance was unrelated to the extent of spider fear, as indicated in the Spider Fear Screening (telephone interview) and the Fear of Spiders Questionnaire (after completion of the experimental task).

^5^Three participants (two phobic, one control) were excluded from skin conductance analyses because their difference scores (spider-bird) deviated more than 3 SD from the group mean.

^6^Importantly, group differences cannot simply be explained by a greater level of general anxiety in phobics rather than controls, because we did not find differences between the two groups on the State-Trait Anxiety Inventory (Spielberger et al., 1983) or the Fear Survey Schedule (Wolpe and Lang, 1969).

^7^However, this time, there was a positive link for controls.

^8^This inconsistency can be related to incongruent findings regarding the amygdala-OFC interplay for the regulation of emotion (Rosenkranz et al., 2003; Quirk and Beer, 2006); vs. (Banks et al., 2007).

^9^According to Botvinick et al. (2001), the ACC plays a major role in the detection of conflicts. Other prefrontal areas should be part of a regulatory system that initiates an adequate response, and at the same time, inhibits unintended responses.
